# Prevalence of toxoplasmosis in semi-domesticated and pet cats within and around Bangkok, Thailand

**DOI:** 10.1186/s12917-021-02965-z

**Published:** 2021-07-22

**Authors:** Tawin Inpankaew, Panpicha Sattasathuchana, Chanya Kengradomkij, Naris Thengchaisri

**Affiliations:** 1 Department of Parasitology, Faculty of Veterinary Medicine, Kasetsart University, Bangkok, 10900 Thailand; 2 Department of Companion Animal Clinical Sciences, Faculty of Veterinary Medicine, Kasetsart University, Bangkok, 10900 Thailand

**Keywords:** Bangkok, IFAT, Pet cats, Semi-domesticated cats, Thailand, Toxoplasmosis

## Abstract

**Background:**

Toxoplasmosis is one of the most common parasitic zoonoses worldwide. Cats become infected after ingesting infected tissue cysts. The objective of the present study was to compare the prevalence of toxoplasmosis in pet cats and semi-domesticated cats in the Bangkok metropolitan region. A survey of *Toxoplasma* infection was conducted in 260 cats (median age [range]: 3 years [10 months–10 years]; 155 females and 105 males) by collecting blood samples from 130 client-owned pet cats and 130 semi-domesticated cats within and around Bangkok during 2016–2017 using indirect fluorescence antibody tests. An IgG antibody to *Toxoplasma* antigen ratio of ≥1:100 was considered positive for *Toxoplasma* infection.

**Results:**

The overall prevalence of *T. gondii* in cats was 6.5% (17/260). The prevalence of *T. gondii* in semi-domesticated cats and pet cats was 11.5 and 1.5%, respectively. Semi-domesticated cats aged 1–5 years (14.9%) had a higher prevalence of infection than domesticated cats (1.3%, *p* = 0.002) of the same age. The odds (95% confidence interval [CI]) of having *T. gondii* infection in semi-domesticated cats were 8.34 (1.86–76.29, *p* = 0.0017) times higher than in pet cats. Interestingly, there was an association between *T. gondii* infection according to city ​region (*p* = 0.002). The odds (95% CI) of having *T. gondii* infection in cats living in the inner city were 4.96 (1.03–47.16, *p* = 0.023) times higher than cats living in the suburb and the vicinity.

**Conclusions:**

The present study identified a higher prevalence of *Toxoplasma* infection in semi-domesticated cats compared with pet cats. The semi-domesticated cats could serve as a zoonotic reservoir. Public health regulations should be implemented to prevent toxoplasmosis spread.

## Background

*Toxoplasma gondii* is a zoonotic protozoan parasite with a worldwide distribution. It is capable of infecting all warm-blooded animals, including humans, and is estimated to infect 4 to 77% of the human population [1]. Members of the family *Felidae* (domestic cats and their relatives) serve as definitive hosts, and other warm-blooded animals, including humans, mice, and rats, serve as intermediate hosts [1]. Cats become infected after eating uncooked meat containing tissue cysts or bradyzoites of *T. gondii* [1, 2]. Bradyzoites are released from an infected tissue and transform into merozoites and tachyzoites before undergoing rapid asexual expansion [1, 2]. An in vitro study revealed that toxoplasma sexual development can occur with the presence of linoleic acid [2]. Another in vitro study revealed that oocysts were released in cat feces as quickly as 3–10 days after ingesting tissue cysts of *T. gondii* [3]. Oocysts sporulate in 1–5 days in the environment and become pathogenic. Toxoplasmosis can be diagnosed based on the cats’ history, clinical signs, and a blood test for toxoplasma antibodies. The indirect fluorescent antibody test (IFAT) has been widely used for detecting *T. gondii* in humans and animals [4].

*T. gondii* is ubiquitous in Bangkok and the surrounding areas. Antibodies to *T. gondii* were reported in the sera of 4.8–11.0% of stray cats residing in Bangkok [5, 6] and 8.3% of farm cats residing to the west of Thailand [7]. There are nearly 500 temples in Bangkok [8], and large numbers of semi-domesticated cats are found roaming in public places and monasteries [5]. It is possible that semi-domesticated cats play a significant role as reservoir hosts for *T. gondii* for humans as well as pet cats. An increasing number of families choose to raise cats as their pets because of the minimal inconvenience compared to dogs. Seroprevalence studies of *T. gondii* are important to public health because the number of pet cats at risk of being infected with *T. gondii.* is growing. In addition, antibodies to *T. gondii* in Thailand was 2.6% in women [9], 25.0% in pregnant women [10] and 6.4% in cat owners [11], respectively.

The objective of the present study was to compare the prevalence of toxoplasmosis in pet cats and semi-domesticated cats within and in the vicinity of Bangkok, Thailand, using IFAT. Factors influencing the seroprevalence of *T. gondii*, including patient characteristics (age, breed, and sex), subdistrict, and city zones, were also identified.

## Results

The seroprevalence of *T. gondii* infection among stray and house cats residing in and around Bangkok by patient characteristics is shown in Table 1. The overall prevalence (95% confidence interval [CI]) of stray and pet cats seropositive for *T. gondii* was 11.5% (6.46–19.03%) and 1.5% (1.86–5.56%), respectively (Table 1). The odds (95% CI) of having *T. gondii* infection in semi-domesticated cats was 8.34 (1.86–76.29, *p* = 0.0017) times higher than in pet cats. Although cats aged 1–5 years (8.7%) had a higher prevalence of infection than cats aged > 5 years (4.3%) and cats aged < 1 year (1.9%), it did not reach statistical significance. Semi-domesticated cats aged 1–5 years (14.9%) had a higher infection rate than domesticated cats (1.3%, *p* = 0.002) of the same age. Overall seroprevalence in females (12/155; 7.7%) was higher than in males (5/105; 4.8%), but it did not reach statistical significance. Both male (10.9%) and female (11.9%) semi-domesticated cats had a higher prevalence of *T. gondii* infection compared with both male (0%, *p* = 0.009) and female (2.8%, *p* = 0.035) domesticated cats (Table 1).

**Table 1:** Effects of patient characteristics on seroprevalence of *T. gondii* infection in semi-domesticated and pet cats

Category	Semi-domesticated cats		Pet cats		Total	
	N	No. positive (%)	N	No. positive (%)	N	No. positive (%)
Breed						
DSH	130	15 (11.5)	52	2 (3.8)	182	17 (9.3)
Persia	–	–	48	0 (0)	48	0 (0)^#^
Maine Coon	–	–	7	0 (0)	7	0 (0)
Scottish Fold	–	–	5	0 (0)	5	0 (0)
Mixed	–	–	18	0 (0)	18	0 (0)
Total	130	15 (11.5)	130	2 (1.5)**	260	17 (6.5)
Age group, years						
< 1	34	1 (2.9)	18	0 (0)	52	1 (1.9)
1–5	87	13 (14.9)	74	1 (1.3)**	161	14 (8.7)
> 5	9	1 (11.1)	38	1 (2.6)	47	2 (4.3)
Sex						
Male	46	5 (10.9)	59	0 (0)**	105	5 (4.8)
Female	84	10 (11.9)	71	2 (2.8)*	155	12 (7.7)

Abbreviation:  DSH = domestic short-haired

^*^p < 0.05 vs. semi-domesticated cat

^**^p < 0.01 vs. semi-domesticated cat

^**#**^*p* < 0.05 vs. DSH

*T. gondii* infection was found in 7 out of 15 districts/provinces, or 46.7%. Bangkok’s Noi district had the highest prevalence of *T. gondii* at 33.3% (7/21). Other districts where *T. gondii* infection (number of positive cats) were found include Bang Khen (2), Bang Phlat (2), Chatuchak (2), Lak Si (1), Phaya Thai (2), and Pathum Thani (1) (Table 2). According to city zone, seroprevalence of *T. gondii* infection among semi-domesticated cats was found to be highest in inner city Bangkok (25.0%) followed by the urban fringe (8.0%) and the suburb and the vicinity (2.5%) (Table 3). In contrast, *T. gondii* infection from pet cats was highest in the suburban areas (2.8%), followed by inner city Bangkok (1.9%) and the urban fringe (0%) (Table 3). There was an association between *T. gondii* infection and city ​region surrounding the metropolis of Bangkok (*p* = 0.002). The odds (95% CI) of having *T. gondii* infection among cats living in the inner city were 4.96 (1.03–47.16, *p* = 0.023) times higher than among cats living in the suburb and the vicinity.

**Table 2:** Seroprevalence of *T. gondii* infection in semi-domesticated and pet cats by subdistrict, Bangkok, Thailand

Subdistrict	Semi-domesticated cats		Pet cats		Total	
	N	No. positive (%)	N	No. positive (%)	N	No. positive (%)
Bangkok Noi	10	7 (70.0)	11	0 (0)^**^	21	7 (33.3)
Bang Sue	10	0 (0)^##^	5	0 (0)	15	0 (0)^#^
Chatuchak	10	1 (10.0)^##^	24	1 (4.2)	34	2 (5.9)^##^
Phaya Thai	10	2 (20.0)^#^	13	0 (0)	23	2 (8.7)^#^
Bang Kapi	10	0 (0)^##^	8	0 (0)	18	0 (0)^##^
Bang Khen	10	2 (20.0)^#^	19	0 (0)^*^	29	2 (6.9)^#^
Bang Phlat	10	2 (20.0)^#^	–	–	10	2 (20.0)
Lat Phrao	10	0 (0)^##^	11	0 (0)	21	0 (0)^##^
SaiMai	10	0 (0)^##^	3	0 (0)	13	0 (0)^#^
Don Mueang	10	0 (0)^##^	3	0 (0)	13	0 (0)^#^
Lak Si	10	1 (10.0)^##^	–	–	10	1 (10.0)^#^
Khlong Sam Wa	10	0 (0)^##^	–	–	10	0 (0)^#^
Nong Chok	10	0 (0)^##^	–	–	10	0 (0)^#^
Nonthaburi	–	–	21	0 (0)	21	0 (0)^##^
Pathum Thani	–	–	12	1 (8.3)	12	1 (8.3)^#^

^*^p < 0.05 vs. semi-domesticated cat

^**^p < 0.01 vs. semi-domesticated cat

^#^*p* < 0.05 vs. Bangkok Noi

^##^*p* < 0.01 vs. Bangkok Noi

**Table 3:** Seroprevalence of *T. gondii* infection in semi-domesticated and pet cats by city zone, Bangkok, Thailand

City zone	Semi-domesticated cats		Pet cats		Total	
	N	No. positive (%)	N	No. positive (%)	N	No. positive (%)
Inner city	40	10 (25.0)	53	1 (1.9)^**^	93	11 (11.8)
Urban fringe	50	4 (8.0)^#^	41	0 (0)	91	4 (4.4)
Suburb and the vicinity	40	1 (2.5)^##^	36	1 (2.8)	76	2 (2.6)^#^

^**^p < 0.01 vs. semi-domesticated cat

^#^p < 0.05 vs. inner city

^##^*p* < 0.01 vs. inner city

In the present study, a larger number of semi-domesticated cats were affected with anemia and leukocytosis compared with pet cats (Table 4). The percentage of cats with anemia that were seropositive versus seronegative for *T. gondii* did not differ (Table 4; *p =* 0.808). The percentage of cats with leukocytosis that were seropositive for *T. gondii* was significantly higher than the percentage of those seronegative for *T. gondii* (Table 4; *p* = 0.031).

**Table 4:** Association between seroprevalence of *T. gondii* and anemia or leukocytosis in semi-domesticated and pet cats

Categories	Negative for *T. gondii*		Positive for *T. gondii*	
	N	No. positive (%)	N	No. positive (%)
Anemia				
Semi-domesticated cats	87	17 (19.5)	13	1 (7.7)
Pet cats	125	1 (0.8)^##^	2	0 (0)
Total	212	18 (8.5)	15	1 (6.7)
Leukocytosis				
Semi-domesticated cats	87	42 (48.3)	13	7 (53.8)
Pet cats	125	5 (4.0)^##^	2	0 (0)
Total	212	47 (22.2)	15	7 (46.7)^*^

^*^p < 0.01 vs. negative for *T. gondii*

^##^p < 0.01 vs. semi-domesticated cats

## Discussion

In this study, IFAT was performed to determine the seroprevalence of toxoplasmosis in semi-domesticated and pet cats within and in the vicinity of Bangkok. The results showed that the overall infection rate was 6.5%. Previous research has indicated that the worldwide distribution of *T. gondii* in cats varies between 6.0 and 74.0% [1], and studies in Asia have shown seropositive rates between 2.2 and 62.8% [5, 6, 11–29] (Table 5). The level of seroprevalence found in the present study was noticeably lower than in other studies in Thailand [5, 11, 26–28]. These variations in seroprevalence rates may have been due to the difference in serological techniques used, the timing of the studies, the sample size, and the varying environmental and management conditions in different parts of the world [30, 31]. The prevalence of toxoplasmosis in semi-domesticated cats (11.5%) was significantly higher than in pet cats (1.5%). This finding was consistent with the results of earlier studies [5, 32–34], which determined that the frequency of *T. gondii* infection in stray animals is generally higher than in pets. Thus, the higher percentage of semi-domesticated cats with *T. gondii* infection may relate to the habits of the cats, which can roam freely inside and outside the temples in Thailand. In this circumstance, cats generally defecate in environments shared with humans, leading to widespread environmental contamination with oocysts [30]. Furthermore, the hunting behavior of cats facilitates infection through the consumption of intermediate hosts (rodents or birds). Cats also can be exposed to oocysts on contaminated ground and may become infected via oral route.

**Table 5:** Seroprevalence of *T. gondii* infection in cats reported previously for various Asian countries, including Thailand

Country	Prevalence (%)	Method	Reference
China	25.2	ELISA	[12]
	21.3	MAT	[13]
Indonesia	59.4	IH	[14]
Iran	35.3	MAT	[15]
Japan	6.0	LAT	[16]
	5.4	LAT	[17]
Korea	15.3	ELISA	[18]
	15.8	ELISA	[19]
	2.2	ELISA	[20]
Malaysia	14.5	IFAT	[21]
Myanmar	41.3	ELISA	[22]
Pakistan	60.0	LAT	[23]
Saudi Arabia	62.8	ELISA	[24]
Singapore	30.7	ELISA	[25]
Thailand	7.3	Sabin-Feldman dye test	[11]
	11.0	LAT	[5]
	4.8	Sabin-Feldman dye test	[6]
	10.1	MAT	[26]
	9.0	IFAT	[27]
	18.7	MAT	[28]
	6.5	IFAT	Present study
Vietnam	72.3	LAT	[29]

Abbreviations: *ELISA* enzyme-linked immunosorbent assay; *IFAT* indirect fluorescence antibody test; *IH* indirect hemagglutination; *LAT* latex agglutination test; *MAT* modified latex agglutination test

In our study, the overall seroprevalence of *T. gondii* infection did not significantly differ between male and female cats, and this result was in agreement with previous reports in Japan [16], Brazil [35], and Saudi Arabia [33]. On the other hand, a higher prevalence of *T. gondii* infection in female cats has been found in Hungary [36] and Poland [37], whereas male cats were reported to have a significantly higher prevalence of *T. gondii* infection (*p* < 0.05) in Norway [38] and Albania [39].

In addition, the seroprevalence of *T. gondii* in semi-domesticated cats in our study was highest in domestic short-haired (DSH) cats (11.5%), females (11.9%), and cats aged 1–5 years (14.9%). In pet cats, the seroprevalence of *T. gondii* was highest in DSH cats (3.6%), females (2.3%), and cats aged more than 5 years (2.6%). This probably relates to the fact that older animals are more likely to have contact with the parasite than younger ones, having higher probabilities of being exposed throughout the years that may increase the chances of infection and contribute to the spread of the oocysts in the environment [31, 40].

The seroprevalence of *T. gondii* was highest among cats residing in inner city Bangkok (11.8%) compared with the urban fringe (4.4%) as well as the suburban and surrounding areas (2.6%). This result was in contrast to previous studies that found *T. gondii* seroprevalence did not differ between cats in urban, semi-urban, and rural areas [41]. Semi-domesticated cats living in monasteries are not regularly dewormed, and most cats are in poor health because they do not have real owners. Moreover, these areas tend to lack proper management of cat feces. In many cases, *T. gondii* infection is asymptomatic in animals, and the only confirmation of infection is the presence of specific anti-*T. gondii* antibodies. Animal sera are generally tested with a commercially available latex agglutination test, modified agglutination test, or IFAT based on native antigens. *T. gondii* antibodies are only indication of previous contact with the parasite which could not be present in the host at the time of the serological analysis, especially IgG to tachyzoites [42]. *Hammondia hammondi* and *Neospora caninum* experimentally present cross-reactivity to *T. gondii* by various serological assays [43]. Additionally, the cross-reactivity between *N. caninum* and *T. gondii* was confirmed by a proteomic study [44]. A study of *T. gondii* and *N. caninum* in Thailand noted that approximately 6% of antibody detection was seropositive in both agents [45]; however, cross-reactivity was uncommon [46].

A limitation of this cross-sectional observational study is that questionnaires to assess cat habitat information may be problematic, particularly regarding exposure to soil, type of diet, consumption of undercooked meat, access to hunting, and information about free roaming or outdoor access—factors that might be related to toxoplasmosis. In addition, the release of oocysts to the environment from cats has not yet been well defined and require further study especially from the cat litter. Moreover, prevalence and the role of *H. hammondi*, an avirulent relative of *T. gondii*, in Thailand has not yet been reported. Thus, epidemiological studies of *H. hammondi* in Thailand are warranted.

These results on toxoplasmosis among cats in Bangkok and the surrounding area are beneficial to researchers, health workers, veterinarians, and policymakers. Urgent attention is required to educate and inform people to increase awareness about toxoplasmosis and risk factors associated with *T. gondii* infection in humans and animals. Furthermore, control measures such as consistent use of antiprotozoal medications, careful disposal of feline feces, and use of disinfectant (l% sodium hypochlorite or 70% ethanol) in living areas if they become contaminated with cat feces are suggested.

## Conclusion

The present study identified a higher prevalence of *T. gondii* infection in semi-domesticated cats compared with pet cats. Therefore, cats in temple communities pose a potential zoonotic risk to humans for transmission of *T. gondii*, and public health regulations should be implemented to prevent toxoplasmosis spread in this population. Further studies in additional areas will be necessary to understand the overall epidemiological status of toxoplasmosis in household and semi-domesticated cats in Thailand.

## Methods

### Animals

**Fig. 1:**
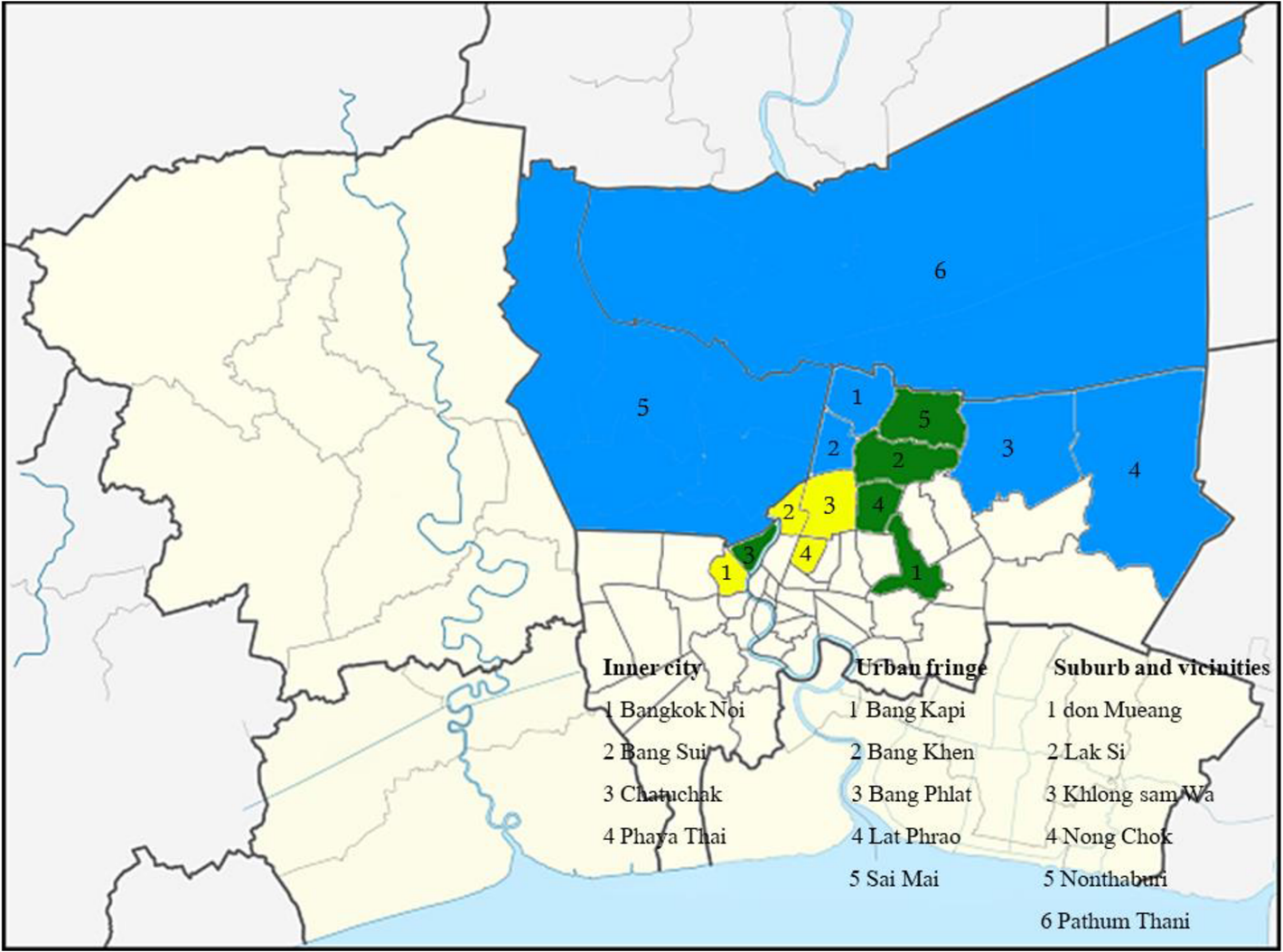
Study areas in and around Bangkok, Thailand. Yellow indicates inner city, green indicates urban fringe, and blue indicates suburb and the vicinity

The sample-collection protocols were reviewed and approved by the Animal Care and Use Committee at Kasetsart University (ACKU60-VET-032). Informed owner consent forms were signed before samples were collected. A total of 260 cats (130 semi-domesticated cats and 130 pet cats) from 13 selected districts in Bangkok and two nearby areas (Fig. 1) were enrolled in the *T. gondii* survey.

The age, sex, and breed of the cats were recorded. A general physical examination was performed for all cats. Approximately 3 ml of blood was collected via the jugular vein and tested for complete blood count (hematocrit [packed-cell volume], mean corpuscular hemoglobin concentration, mean corpuscular volume, white blood cell count, neutrophil count, lymphocyte count, monocyte count, and eosinophil count) to detect anemia and leukocytosis.

### Detection of antibodies to T. gondii using IFAT

An indirect fluorescent antibody test (IFAT) for detection of antibodies to *T. gondii* was performed as previously described [27]. Briefly, tachyzoites of *T. gondii* (RH strain) were maintained using African green monkey kidney (Vero) cells in the minimum essential medium (Life Technologies Corporation, New York, USA) at 37 °C in a 5% CO_2_ air environment. They were harvested and diluted into the concentration of 10^6^ tachyzoites/ml. Then 12 well microscope slides were coated with 10 μl/well of tachyzoites and dehydrated by air-drying at room temperature. The coated microscope slides were then fixed with cold acetone before storing at -20 °C for later use. Each cat serum sample was diluted to 1:100 [47] in phosphate buffered saline (PBS) with 4% bovine serum albumin (Sigma-Aldrich, USA), placed onto the coated antigen slides and incubated at 37 °C for 30 min. Then slides were washed with PBS three times and incubated with a 10 μl/well of caprine anti-feline IgG fluorescein isothiocyanate conjugate (VMRD, Washington, USA) at 37 °C for 30 min. After incubation with secondary antibody conjugate, the slides were washed again with PBS three times, covered with cover slips, and examined under a fluorescence microscope. *T. gondii* positive and negative control sera were used from IgG FA Positive and FA Negative Control, feline origin (VMRD, Washington, USA).

### Statistical methods

Characteristics of individual cats, including sex, age, breed, type of cat, and zone, were analyzed in relation to seroreactivity to identify putative risk factors associated with cat exposure to *T. gondii*. The relationship between the seropositivity and possible associated factors was tested with the Chi-square (χ2) or Fisher’s exact test using STATA version 14.2, and *p*-value of ≤0.05 was considered statistically significant.

## Data Availability

The data used and/or analyzed in the present study are available from the corresponding author on reasonable request.

## Abbreviations

CI: Confidence interval
DSH: Domestic short-haired
ELISA: Enzyme-linked immunosorbent assay
IFAT: Indirect fluorescence antibody test
IH: Indirect hemagglutination
LAT: Latex agglutination test
MAT: Modified latex agglutination test
PBS: Phosphate buffered saline
